# The ratio of cervical lordosis to C7 slope represents the reciprocal change between cervical sagittal alignment and global spinal alignment

**DOI:** 10.1186/s13018-023-03602-1

**Published:** 2023-02-24

**Authors:** Dong-Fan Wang, Shi-Bao Lu, Xiang-Yu Li, Bin Shi, Cheng-Xin Liu, Chao Kong

**Affiliations:** 1 Department of Orthopedics, Xuanwu Hospital, Capital Medical University, No.45 Changchun Street, Xicheng District, Beijing, 100053 China; 2 National Clinical Research Center for Geriatric Diseases, No.45 Changchun Street, Xicheng District, Beijing, China; 3 Beijing Clinical Research Center for Geriatric Diseases, No.45 Changchun Street, Xicheng District, Beijing, China

**Keywords:** Cervical alignment, Global sagittal alignment, Cervical spine, Sagittal balance

## Abstract

**Purpose:**

This retrospective cross-sectional study investigated variations in the ratio of cervical lordosis to C7 slope (CL/C7S) at different stages of global sagittal balance to better understand how global sagittal alignment affects cervical alignment.

**Methods:**

A total of 255 patients with the degenerative lumbar disease were retrospectively studied within a single medical center. Whole spine radiographs were used to evaluate sagittal parameters, mainly including occiput-C2 lordosis (OC2), cervical lordosis (CL), C7 slope (C7S), the ratio of cervical lordosis to C7 slope (CL/C7S), cervical sagittal vertical axis (CSVA), thoracic kyphosis (TK), lumbar lordosis (LL), pelvic tilt (PT), pelvic incidence (PI), PI minus LL mismatch (PI–LL), and sagittal vertical axis (SVA). Patients were divided into the balance group (SVA < 50 mm, PI–LL ≤ 10°), hidden imbalance group (SVA < 50 mm, PI–LL > 10°), and imbalance group (SVA > 50 mm).

**Results:**

Significant correlations were found between CL/C7S and OC2 (*r* = − 0.334), CSVA (*r* = − 0.504), PI–LL (*r* = 0.189), and SVA (*r* = 0.309). Multivariable linear regression analysis indicated that patients in the hidden imbalance group had lower CL/C7S than those in the balance group (*B* = − 0.234, *P* < 0.001), whereas the value of CL/C7S in patients with imbalanced sagittal alignment was higher than those with balanced alignment (*B* = 0.164, *P* = 0.011). The mean value of CL/C7S was 0.71, 0.51, and 0.97 in the balance, hidden imbalance, and imbalance groups, respectively. The global spine tended to tilt forward as the LL decreased, while TK, PT, PI–LL, and SVA increased (all, *P* < 0.001) from the balance stage to the imbalance stage.

**Conclusions:**

CL/C7S tended to be lower when the thoracic extension increased to maintain global sagittal balance at the hidden imbalance stage. Inversely, CL/C7S increased significantly when the global spine showed severe anterior malalignment.

**Supplementary Information:**

The online version contains supplementary material available at 10.1186/s13018-023-03602-1.

## Introduction

The physiologic spine alignment adjusts to maintain a minimum of energy expenditure, with multivariable significant correlations between each of the spinal segments [1]. As the most complex segment of the spine, the cervical spine not only supports the mass of the head but also plays an important role in compensating for thoracolumbar malalignment to keep a horizontal gaze [2]. Many previous studies investigated the correlation between cervical sagittal alignment and global spine alignment. Yu et al. [3] measured sagittal parameters in 120 asymptomatic individuals and reported that the percentages of cervical lordosis (CL) in Roussouly Type 1 and 4 were 60 and 62.5%, while straight cervical alignment accounted for 38.1 and 64.3% in Roussouly Type 2 and 3. Diebo et al. [4] investigated sagittal alignments of 577 patients with thoracolumbar degenerative diseases and indicated that cervical alignment was significantly more lordotic when sagittal vertical axis (SVA) > 50 mm. In other words, the thoracolumbar curves and the global spinal balance have a combined effect on the cervical alignment.

Degenerative lumbar disease (DLD) is a common cause of global spinal malalignment, which is characterized by loss of lumbar lordosis (LL) and anterior translation of SVA. Meanwhile, thoracic extension and pelvic retroversion are two important compensatory mechanisms to maintain sagittal balance of the global spine. Based on the severity of the imbalance, the degeneration can be categorized into three stages: balanced, balanced with compensatory mechanisms (or hidden imbalanced), and imbalanced [5]. During this process, CL was shown to be increased to maintain a horizontal gaze as mentioned before, regardless of whether SVA was within the normal range of values [2]. However, this result might neglect the effect of decreased thoracic kyphosis (TK) on CL and oversimplify the reciprocal change of cervical curvature with the global spinal alignment.

So far, there is rare information on how cervical alignment changes with the degeneration of global sagittal alignment. We hypothesize that the cervical alignment varies between the stages mentioned before. Among all the sagittal parameters, CL is most closely associated with the morphology of the cervical spine [2], while T1 slope (T1S) is an essential variable linking both the cervical sagittal alignment and upper thoracic alignment [6, 7]. It is reasonable to speculate that the ratio of CL to T1S (CL/T1S), which represent the adaptation between the cervical and thoracolumbar spine, might be more appropriate in investigating the reciprocal change between cervical sagittal alignment and global spinal alignment. Considering that the superior endplate T1 could be challenging to visualize on radiographs due to overlying anatomical structures, the C7 slope (C7S) was used to substitute for T1S in this study [8, 9]. Thus, we utilized the ratio of CL to C7S (CL/C7S) to describe the cervical sagittal alignment in this study and aimed to investigate the variations in CL/C7S with the degeneration of global spinal alignment.

## Methods

### Patient population

Under approval from the ethics committee of our hospital, a retrospective study of consecutive patients with DLD from October 2018 to April 2021was performed. Three pathologies were included in this study: lumbar disc herniation, lumbar disc degeneration, and degenerative spondylolisthesis [10]. Other inclusion criteria were: (1) age > 18 years; (2) without malignancy, neurologic disorder, or posttraumatic myelopathy; (3) without presenting with neck pain; (4) no thoracolumbar deformities such as spinal scoliosis or hyperkyphosis caused by vertebral fractures [11]; (5) no history of spine surgery.

### Radiological data acquisition

**Fig. 1:**
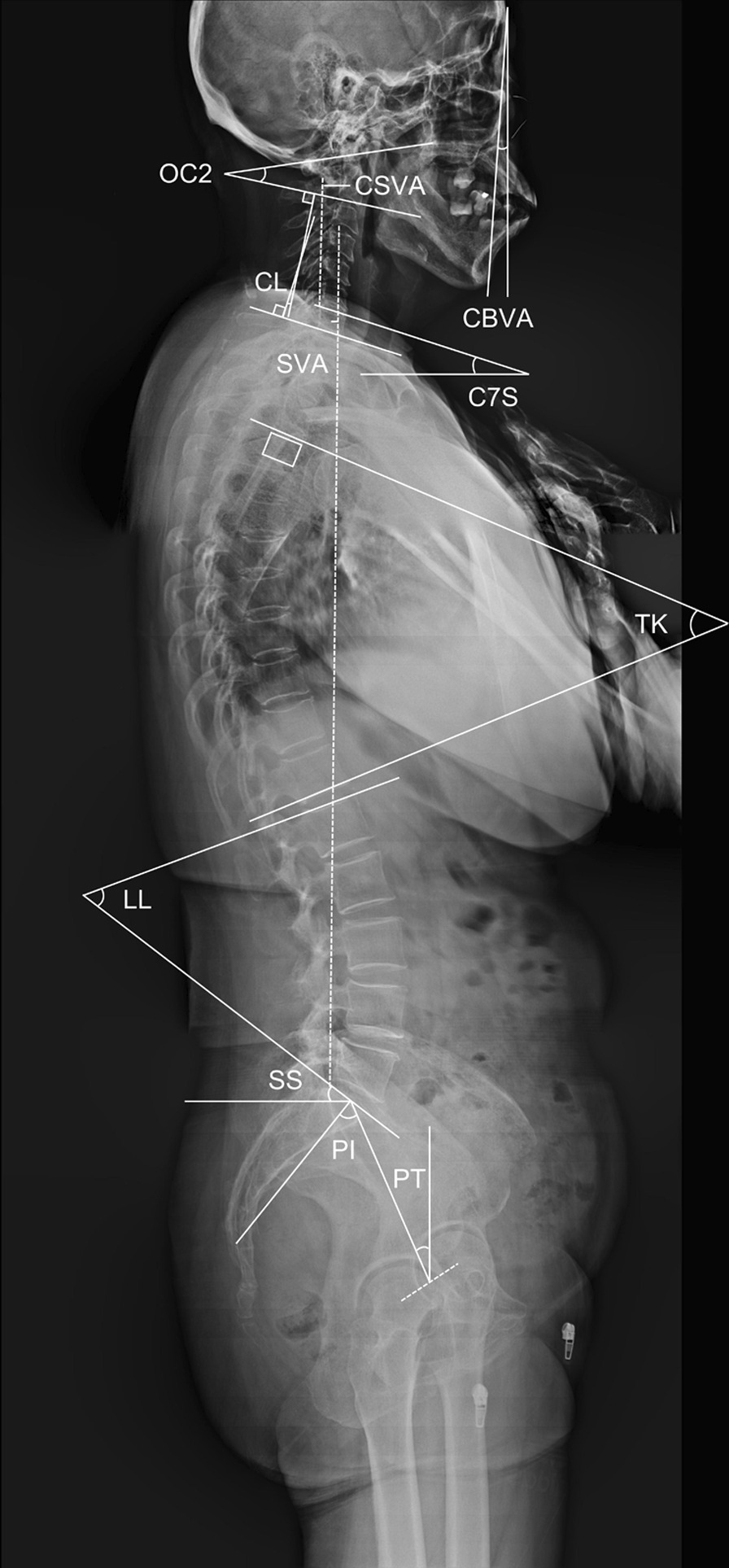
Measurements of spinal sagittal parameters utilized in this study

Whole spine radiographs were used to evaluate spinal sagittal parameters, including: (1) occiput-C2 lordosis (OC2), measured from the McGregor line to the inferior endplate of C2; (2) CL, measured from the lower plate of C2 to the lower plate of C7; (3) C7S, measured from the horizontal line to the superior endplate of C7; (4) cervical sagittal vertical axis (CSVA), the horizontal distance between the C2 plumbline and the posterior-superior corner of C7; (5) TK, measured from the superior endplate of T4 to the inferior endplate of T12; (6) lumbar lordosis (LL), measured from the superior endplate of L1 to the upper endplate of S1; (7) sacral slope (SS), measured from the sacral plate to the horizontal plane; (8) pelvic tilt (PT), measured from the line connecting the midpoint of the superior endplate of S1 to the axis of the femoral heads to the gravity line; (9) pelvic incidence (PI), the angle subtended by the line drawn from the hip axis to the center of upper sacral endplate and the line perpendicular to upper sacral endplate; and (10) SVA, the horizontal distance between the C7 plumbline and the posterior-superior corner of the sacrum. The standing posture was standardized: patients were instructed to stand straightly with a horizontal gaze and hands touching the contralateral collar bones. A horizontal gaze was defined as − 10° ≤ chin-brow to vertical angle (CBVA) ≤ 10° [2]. Measurements of sagittal parameters were illustrated in Fig. 1.

**Fig. 2:**
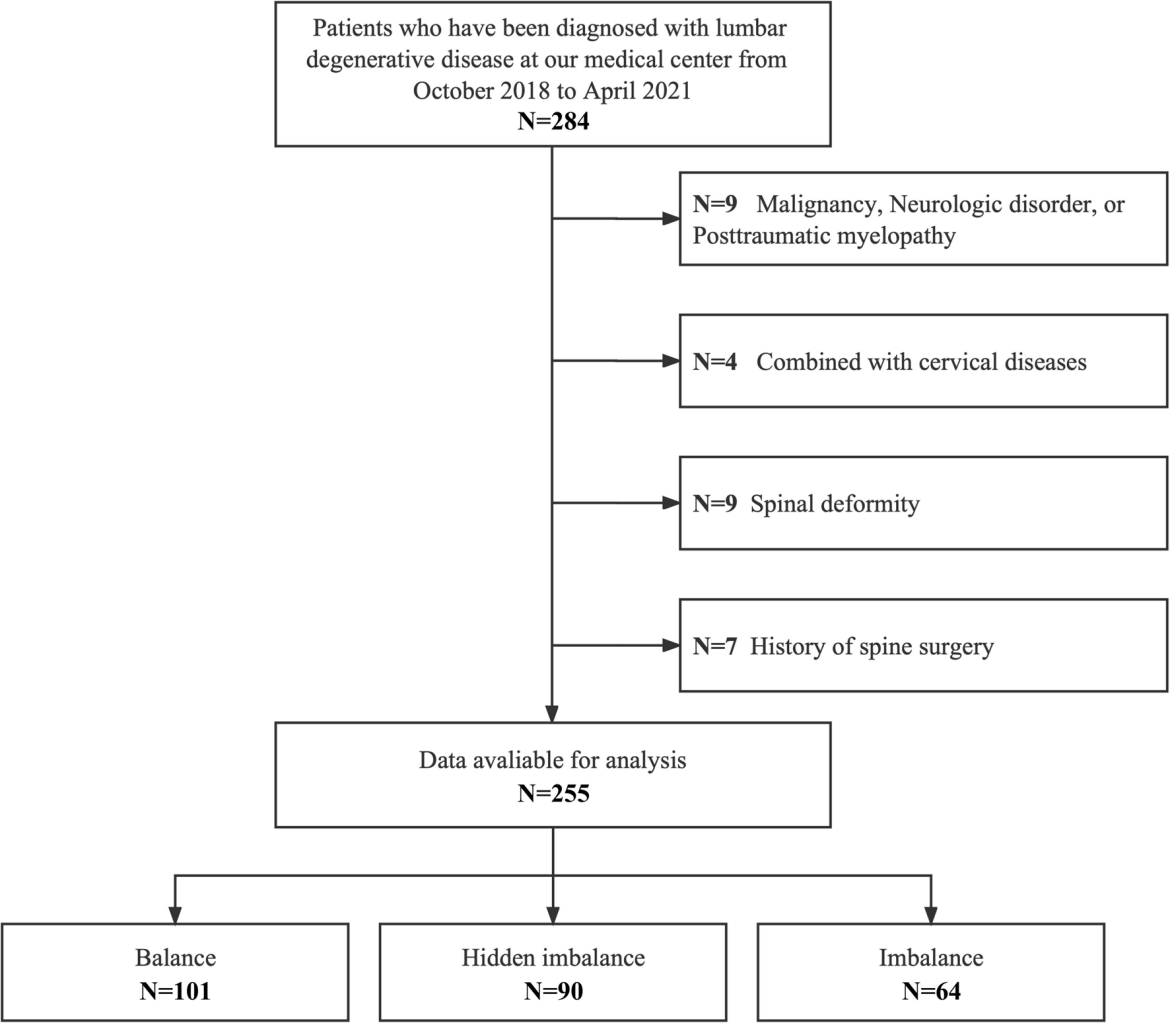
The logic route of the total research

All radiographic data measurements were performed by two trained spinal surgeons (Shi and Liu), and the average of two measurements was taken as the final result. Figure 2 illustrates the flow chart of this study.

### Groups

Previous studies demonstrated the essential roles of PI minus LL mismatch (PI–LL) and SVA paly in assessing spinal sagittal alignment and predicting health-related quality of life [12–14]. Patients were grouped into three groups according to PI–LL and SVA: Balance group (SVA < 50 mm, PI–LL ≤ 10°), Hidden imbalance group (SVA < 50 mm, PI–LL > 10°), and Imbalance group (SVA > 50 mm).

### Statistical analysis

All data were presented as mean value ± standard deviation. Normality was tested by the Shapiro‒Wilk Normality test. Association between CL/C7S and other sagittal parameters were tested using partial correlation controlling for age, CBVA, and PI with a significance level of *P* < 0.00625 (0.05/8). Univariate and multivariable linear regression analyses were conducted to identify influence factors of CL/C7S. Categorical variables were represented as sets of dummy variables. Variables with *P* < 0.05 on univariate analysis were included in the subsequent multivariable linear regression analysis. Continuous variables were compared among groups using the one-way ANOVA and the Kruskal‒Wallis test with Bonferroni or Tamhanes T2 post hoc analysis. The chi-square test was used to compare categorical variables among groups. Intraclass correlation coefficients (ICC) for all parameters were calculated to evaluate the reliability of the intrinsic variability of radiographic measurements. Values of ICC less than 0.5, between 0.5 and 0.75, between 0.75 and 0.9, and greater than 0.90 are indicative of poor, moderate, good, and excellent reliability, respectively.

Data were analyzed using SPSS Statistics (Version 26.0, IBM Corp., Armonk, NY, USA). Statistical significance was set at a level of *P* < 0.05.

## Results

### Baseline characteristics

In total, 255 patients (90 males, 165 females) were enrolled in the present study, with a mean age of 64.55 ± 9.24 years. The baseline data of the whole cohort are summarized in Table 1. All measurements presented high reliability, as the values of ICC > 0.8. The mean value of CL and C7S was 16.26° ± 9.54° and 23.14° ± 6.28°, respectively. The average CL/C7S ratio was 0.70 ± 0.42. In terms of the spinopelvic parameters, LL was 35.15° ± 12.99°, PI was 49.59° ± 9.02°, PI–LL was 14.43° ± 12.04°, and SVA was 27.72 mm ± 44.17 mm on average.

**Table 1:** Baseline characteristics of the whole cohort

Variables	Value
*Demographic parameters*	
Age (years)	64.55 ± 9.24
Female [*n* (%)]	165 (64.7%)
Male [*n* (%)]	90 (35.3%)
*Cervical parameters*	
CBVA (°)	0.13 ± 6.10
OC2 (°)	27.74 ± 8.79
CL (°)	16.26 ± 9.54
C7S (°)	23.14 ± 6.28
CL/C7S	0.70 ± 0.42
CVSA (mm)	18.28 ± 11.32
*Spinopelvic parameters*	
TK (°)	− 32.93 ± 11.42
LL (°)	35.15 ± 12.99
SS (°)	30.48 ± 9.24
PT (°)	19.26 ± 8.28
PI (°)	49.59 ± 9.02
PI–LL (°)	14.43 ± 12.04
SVA (mm)	27.72 ± 44.17

*CBVA* chin-brow to vertical angle; *OC2* occiput-C2 lordosis; *CL* cervical lordosis; *C7S* C7 slope; *CL/C7S* the ratio of cervical lordosis to C7 slope; *TK* thoracic kyphosis; *LL* lumbar lordosis; *SS* sacral slope; *PT* pelvic tilt; *PI* pelvic incidence; *PI–LL* pelvic incidence minus lumbar lordosis mismatch; *SVA* sagittal vertical axis

### Correlation between CL/C7S, cervical parameters, and spinopelvic parameters

The results of the partial correlation analysis are exhibited in Table 2. Significant associations were found between CL/C7S and OC2 (*r* = − 0.334, *P* < 0.001), CSVA (*r* = − 0.504, *P* < 0.001), PI–LL (*r* = 0.189, *P* = 0.003), and SVA (*r* = 0.309, *P* < 0.001). LL was correlated with CL/C7S at the significance level of *P* < 0.05, but the correlation was not significant after Bonferroni’s correction. No correlations were observed between CL/C7S and TK, SS, and PT.

**Table 2:** Partial correlation analysis controlling for pelvic incidence, age, and chin-brow to vertical angle

Variables		CL/C7S
OC2	Coefficient	− 0.334†
	*P*	< 0.001
CSVA	Coefficient	− 0.504†
	*P*	< 0.001
TK	Coefficient	0.092
	*P*	0.146
LL	Coefficient	− 0.169**
	*P*	0.007
SS	Coefficient	− 0.097
	*P*	0.125
PT	Coefficient	0.099
	*P*	0.116
PI–LL	Coefficient	0.189†
	*P*	0.003
SVA	Coefficient	0.309†
	*P*	< 0.001

*OC2* occiput-C2 lordosis; *CSVA* cervical sagittal vertical axis; *TK* thoracic kyphosis; *LL* lumbar lordosis; *SS* sacral slope; *PT* pelvic tilt; *PI–LL* pelvic incidence minus lumbar lordosis mismatch; *SVA* sagittal vertical axis

^**^P < 0.01

^†^*P* < 0.00625 (0.05/8, after Bonferroni’s correction)

### Independent influence factors of CL/C7S

Univariable linear regression analysis showed that OC2 (*B* = − 0.016, *P* < 0.001), CSVA (*B* = − 0.018, *P* < 0.001), LL (*B* = − 0.005, *P* = 0.01), PI–LL (*B* = 0.006, *P* = 0.01), SVA (*B* = 0.003, *P* < 0.001), Hidden imbalance stage (*B* = − 0.198, *P* < 0.001), Imbalance stage (*B* = 0.263, *P* < 0.001) were relevant factors of CL/C7S. Detailed results of the univariable linear regression analysis are shown in the Additional file 1.

The results of the multivariable linear regression analysis are presented in Table 3. Increasing OC2 (*B* = − 0.009; 95% CI [− 0.013 to − 0.004]; *P* < 0.001), higher CSVA (*B* = − 0.017; 95% CI [− 0.021 to − 0.014]; *P* < 0.001) were independent influence factors for superior CL/C7S. Patients in the hidden imbalance group had lower CL/C7S than those in the balance group (*B* = − 0.234; 95% CI [− 0.339 to − 0.128]; *P* < 0.001). The CL/C7S of patients with imbalanced sagittal alignment was 0.164 higher than those with balanced alignment on average (*P* = 0.011).

**Table 3:** The multivariable linear regression analysis

Dependent Variable	Independent Variable	Unstandardized Coefficients (B)	Standardized Coefficients (β)	*t*	*P*	Variance Inflation Factor	*R*	*R*^2^	Adjusted *R*^2^	Durbin- Waston
CL/C7S	Constant	1.352								
	OC2^a^	− 0.009	− 0.180	− 3.705	< 0.001	1.088	0.677	0.459	0.448	1.925
	CSVA^b^	− 0.017	− 0.468	− 9.934	< 0.001	1.020				
	Hidden imbalance stage^c^	− 0.234	− 0.267	− 4.376	< 0.001	1.716				
	Imbalance stage^d^	0.164	0.170	2.574	0.011	2.006				

*CL/C7S* the ratio of cervical lordosis to C7 slope; *OC2* occiput-C2 lordosis; *CSVA* cervical sagittal vertical axis

^a^Increasing OC2 was associated with lower CL/C7S

^b^Increasing CSVA was associated with lower CL/C7S

^c^Patients with hidden imbalanced sagittal alignment had lower CL/C7S than those with balanced sagittal alignment

^d^Patients with imbalanced sagittal alignment had higher CL/C7S than those with balanced sagittal alignment

### Comparison of demographic and radiological parameters among the three groups

The demographic parameters were comparable among groups (Table 4). For cervical parameters, no significant difference in CBVA and CSVA was found in the three groups. The mean value of CL decreased from 15.37° to 11.01° in the hidden imbalance group and then increased to 25.05° in the imbalance group. The average C7S of three groups was 22.10°, 21.47°, and 27.14°, respectively. CL/C7S first decreased from 0.71 to 0.51 and then increased to 0.97 from the balance to the imbalance stage. In terms of spinopelvic parameters, PI was approximate among groups. The global spine tended to tilt forward as the LL and SS decreased, while TK, PT, PI–LL, and SVA increased (all, *P* < 0.001) from the balance stage to the imbalance stage.

**Table 4:** Comparisons of demographic and spinal sagittal parameters among three groups

Variables	Balance (*n* = 101)	Hidden imbalance (*n* = 90)	Imbalance (*n* = 64)	*P*
*Demographic parameters*				
Age (years)	64.18 ± 9.78	63.47 ± 8.90	66.67 ± 8.62	0.092
Male/Female (*n*/*n*)	30/71	35/55	25/39	0.318
*Cervical parameters*				
CBVA (°)	0.74 ± 6.06	− 0.10 ± 5.84	− 0.52 ± 6.50	0.395
OC2 (°)	21.84 ± 8.05^BC^	24.08 ± 8.71^AC^	18.27 ± 9.03^AB^	< 0.001**
CL (°)	15.37 ± 8.93^BC^	11.01 ± 8.2^AC^	25.05 ± 5.21^AB^	< 0.001**
C7S (°)	22.10 ± 5.61^C^	21.47 ± 5.84^C^	27.14 ± 6.30^AB^	< 0.001**
CL/C7S	0.71 ± 0.43^BC^	0.51 ± 0.37^AC^	0.97 ± 0.31^AB^	< 0.001**
CSVA (mm)	18.65 ± 11.73	17.94 ± 10.92	18.19 ± 11.37	0.908
*Spinopelvic parameters*				
TK (°)	− 37.58 ± 9.98^BC^	− 30.57 ± 11.49^A^	− 28.89 ± 11.04^A^	< 0.001**
LL (°)	45.12 ± 8.79^BC^	31.05 ± 9.94^AC^	25.19 ± 11.57^AB^	< 0.001**
SS (°)	33.68 ± 7.96^BC^	29.28 ± 8.89^A^	27.12 ± 10.11^A^	< 0.001**
PT (°)	14.54 ± 5.67^BC^	21.32 ± 7.03 ^AC^	23.81 ± 9.62^AB^	< 0.001**
PI (°)	48.12 ± 8.19	50.44 ± 9.27	50.69 ± 9.73	0.109
PI–LL (°)	2.99 ± 4.46 ^BC^	19.39 ± 7.05 ^AC^	25.51 ± 10.63^AB^	< 0.001**
SVA (mm)	0.12 ± 26.47 ^BC^	15.19 ± 23.07 ^AC^	88.89 ± 27.49^AB^	< 0.001**

*CBVA* chin-brow to vertical angle; *OC2* occiput-C2 lordosis; *CL* cervical lordosis; *C7S* C7 slope; *CSVA* cervical sagittal vertical axis; *TK* thoracic kyphosis; *LL* lumbar lordosis; *SS* sacral slope; *PT* pelvic tilt; *PI* pelvic incidence; *PI–LL* pelvic incidence minus lumbar lordosis mismatch; *SVA* sagittal vertical axis

^A^Significant difference compared to Balance group

^B^Significant difference compared to Hidden imbalance group

^C^Significant difference compared to Imbalance group

**P < 0.01

## Discussion

The sagittal alignment of the cervical spine has been studied extensively in recent decades. It is well-known that the spine functions as a global unit so that the cervical alignment is influenced by the adjacent spinal alignment and the global sagittal balance [2]. In the present study, a novel parameter CL/C7S, which reflects the adaptation between the cervical and thoracolumbar spine, was performed to describe the cervical alignment. Spinal sagittal parameters at the balance, hidden imbalance, and imbalance stages were evaluated to investigate the effect of global sagittal balance on cervical alignment in more detail. Results indicated that CL/C7S first decreased and then increased with the deterioration of spinal sagittal alignment.

At the early stage of degeneration, thoracic extension and pelvic retroversion are two essential mechanisms that compensate for the loss of LL to maintain the sagittal balance [15], [16]. Consistently, we observed that the LL was significantly lower, while TK, PT, and PI–LL were prominent higher in the hidden imbalance group than those in the balance group (Table 4). Concerning the cervical alignment, CL and CL/C7S presented a significant decrease from the balance stage to the hidden imbalance stage (Table 4). It is worth noting that the CL/C7S was positively associated with PI–LL (*r* = 0.189) based on the correlation analysis, but results of multivariable linear regression suggested that CL/C7S was lower in patients with hidden imbalanced alignment than those in a balanced state (*B* = − 0.234). We speculate that the adjacent segments of the cervical spine played a major role in the variation of CL/C7S at the initial stage of global sagittal malalignment. Precisely, the increase of TK led to the decrease of CL, which then contributed to the reduction of CL/C7S. Similarly, In a recent meta-analysis containing 15,364 asymptomatic patients with balanced alignment, Guo et al. [17] also proposed that cervical curvature was inversely correlated with TK. Therefore, the cervical spine adjusted lordosis curvature to adapt to the decrease in spinopelvic curvature, which led to the decrease in CL/C7S. The cervical sagittal alignment became straighter when the global spine was hidden imbalanced despite the SVA increasing.

At the late stage of degeneration, the global sagittal balance deteriorates as the compensatory changes in the thoracic spine and pelvis are insufficient [15]. In this study, though TK and PT increased significantly from the hidden imbalance stage to the imbalance stage, the global spine still presented severe anterior malalignment as SVA increased to 88.89 mm on average (Table 4). Meanwhile, CL and C7S of the imbalance group increased by nearly 14° and 6° compared to that in the hidden imbalance group, respectively, whereas CSVA did not differ significantly between the two groups (Table 4). Results indicated that CL took the leading role in constituting the cervical curvature to maintain the cervical sagittal balance as the line of gravity showed forward translation. In a retrospective study of forty-eight patients without neck pain, Matsubayashi et al. [18] also showed that the lower cervical curvature had an advantageous effect on the adjustment of cervical sagittal translation. Moreover, imbalanced sagittal alignment was correlated with higher CL/C7S according to the multivariable linear regression (*B* = 0.164). Contrary to the variation mentioned before, the curvature of the cervical spine increased when the global spine was imbalanced, which made a positive contribution to maintaining horizontal gaze and cervical sagittal balance. Thus, we speculate that a too high CL/C7S arising from the increase in CL might indicate deterioration of the global sagittal balance. In this study, the value of CL/C7S ranged from 0.61 to 0.81 in the majority of patients with balanced sagittal alignment based on the 95% confidence interval. Malalignment of the global spine should be excluded in patients with CL/C7S higher than 0.81 but no whole spine radiograph. With respect to the spinal alignment, different parts of the spine regulate reciprocally to maintain a horizontal gaze and economic posture [19]. Under the influence of the thoracolumbar spine and the global sagittal balance, the curvature of the cervical alignment decreases firstly to adapt to the variation of thoracolumbar alignment and then increases to maintain horizontal gaze acquisition.

Several limitations still exist in this study. Firstly, the sample size from a single center was relatively limited. Secondly, this cross-sectional comparative study lacks longitudinal data to analyze individual data changes over time. Thirdly, this study described the sagittal alignment of the cervical spine; additional variables in the role of the cervical spine should be considered in future studies, such as head position and paraspinal muscle, chest morphology, and soft tissue. Despite the limitations, this study provided us with a better understanding of how cervical sagittal alignment varies with the development of global spinal degeneration.

## Conclusions

The cervical sagittal alignment, which was represented by CL/C7S, varied with the global sagittal alignment. CL/C7S tended to be lower when the thoracic kyphosis decreased to maintain global sagittal balance. Inversely, CL/C7S increased significantly when the global spine showed severe anterior malalignment. The value of CL/C7S ranged from 0.61 to 0.81 in the majority of patients with balanced sagittal alignment. This study can provide us with a more comprehensive understanding of the interconnectedness between cervical alignment and global spinal alignment.

## Supplementary Information

**Additional file 1**. Univariate linear regression analysis between age, sex, sagittal parameters, global balance state, and CL/C7S.

## Data Availability

The dataset used and/or analyzed during the current study is available from the corresponding author on reasonable request.

## Abbreviations

CBVA: Chin-brow to vertical angle
OC2: Occiput-C2 lordosis
CL: Cervical lordosis
T1S: T1 slope
C7S: C7 slope
CSVA: Cervical sagittal vertical axis
CL/T1S: The ratio of CL to T1S
CL/C7S: The ratio of CL to C7S
TK: Thoracic kyphosis
LL: Lumbar lordosis
SS: Sacral slope
PT: Pelvic tilt
PI: Pelvic incidence
SVA: Sagittal vertical axis
PI–LL: Pelvic incidence minus lumbar lordosis mismatch
PSM: Propensity score matching
DLD: Degenerative lumbar disease
