# Supplementary material for: The ratio of cervical lordosis to C7 slope represents the reciprocal change between cervical sagittal alignment and global spinal alignment
Source: J Orthop Surg Res. 2023 Feb 24;18:138. doi: 10.1186/s13018-023-03602-1 (PMC9951451; doi:10.1186/s13018-023-03602-1)
Supplement: Supplementary file 1 — Additional file 1. Univariate linear regression analysis between age, sex, sagittal parameters, global balance state, and CL/C7S. [file 13018_2023_3602_MOESM1_ESM.docx]

Table. Univariate linear regression analysis between age, sex, sagittal parameters, global balance state, and CL/C7S

|  | CL/C7S | |
| --- | --- | --- |
| Variables | Unstandardized Coefficients (95% CI) | *P* |
| Age | 0.001 (-0.005 to 0.006) | 0.777 |
| Male† | -0.116 (-0.223 to -0.009) | 0.064 |
| CBVA | -0.003 (-0.011 to 0.006) | 0.496 |
| OC2 | -0.016 (-0.021 to -0.010) | <0.001** |
| CSVA | -0.018 (-0.022 to -0.014) | <0.001** |
| TK | 0.003 (-0.001 to 0.008) | 0.165 |
| LL | -0.005 (-0.009 to -0.001) | 0.010* |
| SS | -0.004 (-0.010 to 0.001) | 0.148 |
| PT | 0.004 (-0.002 to 0.010) | 0.179 |
| PI | -0.001 (-0.007 to 0.005) | 0.767 |
| PI-LL | 0.006 (0.001 to 0.010) | 0.010* |
| SVA | 0.003 (0.002 to 0.004) | <0.001** |
| Hidden imbalance† | -0.198 (-0.307 to -0.089) | <0.001** |
| Imbalance† | 0.263 (0.143 to 0.383) | <0.001** |

CBVA, chin-brow to vertical angle; OC2, occiput-C2 lordosis; TK, thoracic kyphosis; LL, lumbar lordosis; SS, sacral slope; PT, pelvic tilt; PI, pelvic incidence; PI-LL, pelvic incidence minus lumbar lordosis mismatch; SVA, sagittal vertical axis.

†, The categorical variable was set as dummy variable.

*, *P* < 0.05

**, *P* < 0.01
